# Risk factors for transient and permanent congenital hypothyroidism: a population-based case-control study

**DOI:** 10.1186/s13044-021-00103-3

**Published:** 2021-05-05

**Authors:** Fariba Abbasi, Leila Janani, Malihe Talebi, Hosein Azizi, Lotfali Hagiri, Shahnaz Rimaz

**Affiliations:** 1 Department of Epidemiology, School of Public Health, Iran University of Medical Sciences, Tehran, Iran; 2 Department of Biostatistics, Faculty of Health, Iran University of Medical Sciences, Tehran, Iran; 3 Prevention and Care of Non-communicable Diseases, Tabriz University of Medical Sciences, Tabriz, Iran; 4 Research Center of Psychiatry and Behavioral Sciences, Tabriz University of Medical Sciences, Tabriz, Iran; 5 Department of Epidemiology and Biostatistics, School of Public Health, Tehran University of Medical Sciences, Tehran, Iran; 6 Department of Epidemiology, School of Public Health, Urmia University of Medical Sciences, Urmia, Iran; 7 Radiology Biology Research Center, Department of Epidemiology, School of Public Health, Iran University of Medical Sciences, Tehran, Iran

**Keywords:** Transient, Permanent, Hypothyroidism, Case control, East Azerbaijan

## Abstract

**Background:**

Congenital hypothyroidism (CH) is the most important cause of mental and physical retardation in newborns. The prevalence of CH has been reported high in East Azerbaijan province of Iran. However, the risk factors for CH are poorly understood. This study aimed to determine and compare risk factors for permanent and transient CH in East Azerbaijan, Iran.

**Methods:**

A case-control study was conducted in the Iranian national screening program for CH. This study included 680 neonates: 340 neonates with confirmed CH and 340 matched healthy controls born at the same period and from the same residential area as the cases. Multiple logistic regression analyses were used to estimate the crude and adjusted odds ratios and 95% confidence intervals for the association between different risk factors and transient and permanent CH.

**Results:**

Out of the 680 participants, 364 (53.53%) were male. Family history of CH (OR = 5.09, 95% CI: 1.66–15.63), neonatal jaundice (OR = 3.89, 95% CI: 2.36–6.43) and parental consanguineous relation (OR = 2.19, 95% CI: 1.51–3.17) were associated with an increased risk of permanent CH. Likewise, the use of Betadine in pregnancy (OR = 4.87, 95% CI: 1.45–16.28), family history of CH (OR = 5.98, 95% CI: 2.04–17.48), neonatal jaundice (OR = 2.81, 95% CI: 1.75–4.52), parental consanguineous relation (OR = 3.86, 95% CI: 1.92–5.74), and gestational age at birth (OR = 3.2, 95% CI: 1.90–5.41) were identified as risk factors for transient CH.

**Conclusion:**

Family history, neonatal jaundice, gestational age at birth, and Betadine usage in pregnancy are associated with CH.

## Introduction

Congenital hypothyroidism (CH) in neonates stems from impaired thyroid hormone synthesis, which can lead to severe mental retardation with inadequate development of the child’s brain, in the absence of timely identification or treatment. Neonatal CH occurs in both transient and permanent forms. The distribution of CH varies from country to country, for example, 1 in 2070 births in the United States [1] and 1 in 2421 live births in China [2]. The average incidence of CH in Fars province, Iran is 1 in 1000 live births [3], 1:748 in Isfahan province [4], while the average incidence rate in East Azerbaijan province of Iran is 1 in 550–600 live births [5]. However, a countrywide review study revealed that the average incidence rate of CH is 2 in 1000 live births in Iran [6].

Evidence suggests the risk factors affecting the incidence of neonatal CH is different in various countries. For example, low birth weight and family history in China [7, 8], congenital anomalies, gender, gestational diabetes, gestational age greater than 40 weeks in Egypt [9], and congenital anomalies and twin pregnancy in Italy [10] have been identified as risk factors for neonatal CH. Moreover, lowering the diagnostic cut-off levels in different CH screening programs could have contributed to an increased prevalence of detected CH. There are different screening cut-off levels in different countries, which can lead to difficulties in comparing the prevalence of CH [7, 11].

There may be risk factors specific to the province that might have contributed to the high prevalence of this disease. Most of the studies conducted in Iran are often descriptive rather than analytical and are based on the city level small-sized samples. Moreover, few studies have focused on the differences between the transient and permanent state of the disease. Therefore, this case-control study aimed to identify and compare important risk factors affecting the incidence of transient and permanent CH in East Azerbaijan province, Iran.

## Method

### Study design

This case-control study was conducted on neonates from East Azerbaijan province who had participated in the national CH screening program. In Iran, medical universities are responsible authorities for the health system in each province. CH screening program is a national program in Iran with national guidelines according to the best available evidence and WHO recommendations. In the national screening program for CH, all neonates are screened within 3–5 days of birth. This program is currently running by first-line health service providers in all cities and villages of the Iranian population. Screening tests are performed on filter paper specimens and then they are sent to referral labs in the center of provinces. All these processes are done in paper form and the statistics are sent from bottom to top [6, 12].

In this study, all neonates, who were born in East Azerbaijan province from the beginning of 2011 to the end of 2013 and were diagnosed with permanent or transient CH following heel prick screening and confirmatory tests, were included in the case group. Based on the province’s health ministry, the total number of neonates with CH was 405. Of those, 65 neonates were excluded due to study criteria, ethical considerations, and the unavailability of the address or telephone number of the patients who migrated, therefore 340 patients whose information was available at the health department of the province that was included in the study. Of 340 neonates, 204 neonates had transient CH and 136 neonates had permanent CH.

### Case subjects

The neonates with thyroid stimulating hormone (TSH) > 20 mU/L in neonatal CH screening on the heel prick screening, and with TSH > 10 mU/L and free thyroxine (T4) < 6.5 μg/dl in venous blood test, who had received levothyroxine for more than 3 years (according to the national guidelines) after confirmation from the focal point program (often overseen by an endocrinologist who is responsible for initiating and ending treatment to prevent error and integrate appropriate case management), were chosen as permanent CH subjects. However, neonates with TSH > 20 mU/L in neonatal CH screening on the heel prick screening, and with TSH > 10 mU/L and T4 < 6.5 μg/dl in venous blood test, who had received levothyroxine for less than 3 years (according to the national guidelines) after confirmation by the focal point specialist, were included as transient CH subjects.

### Control subjects

The infants, who had TSH < 5 mU/L in the heel prick screening or TSH between 5 and 19.9 mU/L in heel prick screening that had reduced to less than 5 mU/L in the re-examination of the heel prick test or less than 10 mU/L in venous blood test, were categorized as healthy control group after matching time of birth and residence with the cases.

### Data collection

Data was collected from the Ministry of Health checklists. Some potential risk factors, such as the use of Betadine (vaginal) during pregnancy by the mother and the presence of jaundice in the neonate, were not available on this checklist. We collected this information from the household records collected at homes and health centers. Betadine usage was defined as the vaginal usage during the pregnancy at least once. Neonatal jaundice was defined as jaundice presenting within 28 days after birth.

Parental consanguineous relationship was defined as any degree of family relationship between the neonate’s mother and father.

The data on healthy controls were collected from the Health Integrated System (SIB) and household records collected at homes and health centers. In case of data unavailability, in-person interviews were conducted.

### Statistical analysis

The Kolmogorov-Smirnov test was used to assess the normality of data distribution. The *t*-test was used to compare the quantitative variables of the control and case groups, if the data were normally distributed. The chi-square test was used to compare the qualitative variables of the case and control groups and data were present as median and quartile 1 and quartile 3. Non-parametric test (Mann-Whitney) was used if the distribution of data was not normal. Each variable in the single-variable analysis that had *P* < 0.2, was analyzed by multiple logistic regression, to evaluate the most important risk factors for transient and permanent CH controlling the effect of confounders, and estimate the adjusted odds ratio (OR). The effects of independent variables on the three-state response variable (permanent, transient, and control) were evaluated using the multiple logistic regression. SPSS software (version 22) was used to analyze the data. For all tests, the error rate was considered to be 5%.

## Results

Overall, this study included a total of 680 participants including 340 neonates with CH in the case group, which consisted of 136 neonates with permanent CH and 204 neonates with transient CH, and 340 healthy neonates. Of all participants, 364 (53.53%) were male. The percentage of newborn boys in the transient CH group was 53.4% (*n* = 109) and 54.4% (*n* = 74) in the permanent CH group. The percentage of newborn boys in the control group was 53.2% (*n* = 181).

### Risk factors associated with transient CH

Table 1 shows the associations between the potential risk factors and the transient CH. According to univariate analysis, parental consanguineous marriage (*P* = 0.001), Betadine usage in pregnancy (*P* = 0.003), history of CH in first-degree relatives (*P* = 0.001), neonatal jaundice (*P* = 0.001), and gestational age (*P* = 0.001) were associated with transient CH.

**Table 1:** Association between transient congenital hypothyroidism (CH) and potential risk factors

Variable	Control ***N*** = 340	Transient CH ***N*** = 204	OR (95% CI)	***P*** value*
**Gestational age (week)**				
≤ 37	310	153	3.44 (2.1–5.62)	0.001
> 37	30	51	1	
**Birth length (cm)**				
≤ 50	100	53	1.18 (0.8–1.75)	0.223
> 50	240	151	1	
**Parental consanguineous marriage**				
No	296 (87.1)	161 (78.9)	1	1
Yes	44 (12.9)	43 (21.1)	2.17 (1.57–3)	0.001
**Betadine usage**				
No	336 (98.2)	191 (93.6)	1	1
Yes	4 (1.2)	13 (6.4)	5.62 (1.8–17.5)	0.003
Gender				
Male	181 (53.2)	109 (53.4)	1	1
Female	159 (46.8)	95 (46.6)	(0.72–1.4)	0.97
History of CH in the first degree				
No	335 (98.5)	188 (92.2)	1	1
Yes	5 (1.5)	16 (7.8)	5.6 (2.02–15.5)	0.001
**Neonatal jaundice**				
No	299 (87.9)	141 (69.1)	1	1
Yes	41 (12.1)	63 (30.9)	3.53 (2.26–5.51)	0.001
**Birth weight (gr)**				
< 2500	19 (5.6)	23 (11.3)	1	1
4000–2500	308 (90.6)	174 (85.3)	0.39 (0.22–0.69)	0.01
> 2500	13 (3.8)	7 (3.4)	0.39 (0.15–1.3)	0.058
**Mother’s age (year)**				
< 20	43 (12.6)	22 (10.8)	1	1
20–30	97 (28.5)	77 (37.7)	1.6 (0.89–2.94)	0.1
30–40	127 (37.4)	78 (38.2)	1.2 (0.36–1.42)	0.54
> 40	73 (21.5)	27 (13.2)	0.74 (0.78–1.45)	0.34

* ***Χ***^**2**^ test was used in the all tests

### Risk factors associated with permanent CH

Parental consanguineous marriage was significantly more frequent in the case group (22.1% versus 12.9% in the control group; *P* = 0.001) (Table 2). More mothers in the case group had used Betadine during pregnancy than those in the control group (4% versus 1.2%, *P* = 0.039). History of CH in first-degree relatives and neonatal jaundice were observed more frequently in the case group than in the control group (*P* = 0.001).

**Table 2:** The relationship between permanent congenital hypothyroidism (CH) and potential risk factors

Variables	Control ***N*** = 340	Permanent CH ***N*** = 136	OR (95% CI)	P value*
**Parental consanguineous marriage**				
No	296 (87.1)	106 (77.9)	1	1
Yes	44 (12.9)	30 (22.1)	2.31 (1.6–3.3)	0.001
**Betadine usage**				
No	336 (98.8)	130 (95.5)	1	1
Yes	4 (1.2)	6 (4.4)	3.8 (1.07–13.8)	0.039
**Sex**				
Boy	181 (53.2)	74 (54.4)	1	1
Girl	159 (46.8)	62 (56.6)	0.95 (0.64–1.42)	0.81
**History of CH in the first degree family**				
No	335 (98.5)	121 (89)	1	1
Yes	5 (1.5)	15 (11)	8.25 (2.93–23.2)	0.001
**Neonatal jaundice**				
No	299 (87.9)	84 (61.8)	1	1
Yes	41 (12.1)	52 (38.2)	4.5 (2.8–7.26)	0.001
**Birth weight (gr)**				
< 2500	19 (5.6)	21 (15.4)	1	1
4000–2500	308 (90.6)	110 (80.9)	0.29 (0.14–0.56)	0.01
> 2500	13 (93.8)	5 (3.7)	0.31 (0.09–1.04)	0.059
**Mother’s age (year)**				
< 20	43 (12.6)	24 (17.6)	1	1
20–30	97 (28.5)	48 (35.3)	1.6 (0.89–3.5)	0.1
30–40	127 (37.4)	41 (30.1)	1 (0.89–2.94)	0.11
> 40	73 (21.5)	23 (16.9)	0.31 (0.57–1.8)	0.93

*Chi-square test was used in the all tests

Table 3 shows the comparison of gestational age and birth length among three groups of participants: permanent CH, transient CH and controls. The median birth length of neonates with both permanent and transient CH was 49 cm as compared to 50 cm in healthy infants (*P* = 0.002). Additionally, the median gestational age at birth in both permanent and transient CH group was 38 weeks as compared to 39 weeks in the control group and it was a significant difference (*P* = 0.003).

**Table 3:** Comparison of gestational age and birth length among controls, permanent and transient hypothyroidism

Variable	Permanent CH		Transient CH		Control		*P* value^a^ One way ANOVA
	Median	(Q25, Q75)	Median	(Q25, Q75)	Median	(Q25, Q75)	
**Birth length (cm)**	49	(47–50)	49	(48–51)	50	(48–51)	0.002
**Gestational age (week)**	38	(38–40)	38	(37.2–39)	39	(38–40)	0.003

*Q* Quartile

^a^*P-*value indicates a significant mean difference among study groups

### Multiple logistic regression analysis

Table 4 shows the results of multiple logistic regression analysis to assess the effects of different potential risk factors for permanent and transient CH.

**Table 4:** The results of multiple logistic regression estimation of effects of risk factors for transient congenital hypothyroidism (CH) and permanent congenital hypothyroidism (CH)

VARIBLE	Comparison of ***permanent*** CH and controls (Adjusted OR, 95% CIs)	Comparison of ***transient*** CH and controls (Adjusted OR, 95% CIs)
**Parental consanguineous marriage**		
No	1	1
Yes	2.19 (1.51–3.17)	3.86 (1.92–5.74)
***P value***	0.001	0.001
**Betadine usage in pregnancy**		
No	1	1
Yes	2.9 (0.93–11.4)	4.87 (1.45–16.28)
***P*** **value**	0.135	0.01
**History of CH in the first degree**		
No	1	1
Yes	5.09 (1.66–15.63)	5.98 (2.04–17.48)
***P*** **value**	0.004	0.001
**Neonatal jaundice**		
No	1	1
Yes	3.89 (2.36–6.43)	2.81 (1.75–4.52)
***P*** **value**	0.001	0.001
**Birth length**		
No	1	1
Yes	1.2 (0.73–1.97)	0.99 (0.65–1.53)
***P*** **value**	0.465	0.685
**Gestational age**		
No	1	1
Yes	1.47 (0.76–2.84)	3.20 (1.90–5.41)
***P*** **value**	0.247	0.001

The use of Betadine during pregnancy (OR = 4.87, 95% CI: 1.45–16.28), history of CH in the first-degree relatives (OR = 5.98, 95% CI: 2.04–17.48), neonatal jaundice (OR = 2.81, 95% CI: 1.75–4.52), and parental consanguineous marriage (OR = 3.86, 95% CI: 1.92–5.74), were associated with an increased risk of transient CH. Moreover, increased gestational age at birth was also associated with transient CH (*P* = 0.001), however, we did not found a significant association between gestational age and permanent CH (*P* = 247).

Regarding the permanent CH, history of CH in the first-degree relatives (OR = 5.09, 95% CI: 1.66–15.63), neonatal jaundice (OR = 3.89, 95% CI: 2.36–6.43), and parental consanguineous marriage (OR = 2.19, 95% CI: 1.51–3.17) were associated with an increased risk of permanent CH (Table 4).

## Discussion

This case-control study was performed to identify the risk factors for CH as a multifactorial disease in East Azerbaijan province. In this study, the incidence rate of CH from the beginning of 2011 to the end of 2013 was 1 in 545 live births. In comparison, similar studies around the world have reported an incidence of 1 in 2020 live births in Egypt [9], 1 in 2700 live births in Italy [10], and 1 in 3447 live births in Australia [13]. Therefore, it seems that the incidence rate of CH in East Azerbaijan Province is higher than in other countries. In Iran, a systematic review and meta-analysis study across all provinces revealed that the incidence rate of CH is 2 per1000 (ranged 1 to 3 in 1000) live births [6].

It is possible that high rates of consanguineous marriage with an associated risk of hereditary thyroid hormone deficiency and ethnicity are important factors in increasing the incidence of CH in this province [14, 15]. The cut-off levels used in different screening programs may also influence the incidence rate [16, 17]. For example, the incidence of permanent CH in Greece [18] was reported at 1:1758 and 1:2441 with TSH cut-off levels of 10 and 20 mU/L, respectively.

Throughout Iran, a cut-off level of TSH = 10 mU/L is used for screening [19]. Therefore, it seems that the higher incidence of CH in East Azerbaijan province is attributed to other genetic and environmental factors. In this study, we found that almost 60% neonates with CH had transient CH. These results suggest neonates with CH should be followed up for a longer period to rule out transient CH. However, most studies have only included permanent CH patients [10, 20].

This study showed that history of CH in first-degree relatives, consanguineous marriage, neonatal jaundice, and birth height is associated with an increased risk of CH. Moreover, in transient CH, in addition to the above-mentioned risk factors, the use of Betadine in the pregnancy and the gestational age are also associated with an increased CH risk.

One of the most important risk factors identified in this study and other national studies [6, 21] is the history of CH in first-degree relatives and consanguineous marriage. The frequency of history of CH in relatives was 11.5% in the case group and 1.5% in the control group. This risk factor had the highest OR for both transient and permanent CH, which is in line with the other studies from Iran [15, 22] as well as other countries [10]. Previous studies have shown that the risk of CH is higher in Asian families than in other races [1], which may be related to a higher prevalence of consanguineous marriage. These results support the evidence for the strong effect of the genetic factors in the pathogenesis of transient and permanent CH. The genetic disorders causing hypothyroidism, such as dyshormonogenesis, show an autosomal recessive transmissions pattern [23]. Therefore, a history of thyroid disease in other family members can be one of the main risk factors for CH, and it is important to pay attention to family history in pregnant mothers.

In the present study, 30.9% of the neonates with transient CH and 38.2% of the neonates with permanent CH (as compared to 11.3% healthy controls) suffered from neonatal jaundice. Other similar studies have also reported a similar association between neonatal jaundice and CH [24].

This study, consistent with the findings of a previous study [25], showed a significant association between the birth length and the incidence of transient and permanent CH. The incidence of permanent and transient CH was higher amongst neonates with short height as compared to neonates with normal height. Khammarnia and colleagues also showed similar results with a two-fold increased risk of CH among children with short height than those with normal height [26].

In the present study, 53.5% of neonates with transient CH were boys and 46.5% girls, with male to female ratio of 1.14. In permanent CH, male to female was 1.19 (54.5% boys and 45.5% girls). In our study, there was no significant difference between male and female sex among neonates with CH. Zainalzadeh et al. also found no gender difference in the incidence of CH in a study from East Azerbaijan province [27]. In most case-control studies, the sex factor has been included as a matched variable [28]. However, other studies indicate the role of the female gender as a risk factor for CH [29, 30].

In this study, there was no relationship between maternal age and transient or permanent CH, similar to previous studies showing a lack of relationship between mother’s age and CH [14]. In contrast, Khammarnia and colleagues [26] showed that infants born to young mothers were at an increased risk of CH, Harris et al. found that infants born to mothers aged 40 years or older had an increased risk of CH [1].

This study showed no significant association between birth weight and transient or permanent CH, as shown by Khammarnia et al. previously [26].

In our study, Betadine usage in the pregnancy was a risk factor for transient CH. Other studies from Iran have shown similar results [14]. Studies have shown that iodine (present in Betadine) has an inhibitory effect on thyroid hormone secretion [31]. The use of iodine-containing substances during pregnancy can be absorbed into the mother’s blood and secreted in breast milk. Therefore, newborns are more exposed to iodine through the breast milk [32]. Use of iodine-containing substances, such as vaginal Betadine, due to rapid absorption through the skin and mucosa, and immediately crossing through the placenta, can cause an increase in iodine level in mothers and, consequently, in newborns [32].

This study also showed an association between gestational age at birth and CH. Previous studies have examined the relationship between CH and gestational age at birth. Medda et al in a study from Italy showed that only a gestational age at birth above 41 weeks to be significantly associated with CH [33]. In the study by Abedi et al [34], gestational age at birth, after controlling the effects of other variables in multiple regerssion analysis, was not significantly associated with permanent CH; however, gestational age at birth was found to be a risk factor for transient CH.

## Limitations

A limitation of this study is selection bias due to the information for patients from Sarab city in the province was not accessible and therefore had to be excluded. Another concern is random error; however, because of our large sample size as compared to the previous studies, it is likely that this bias is small. Additionally, in order to reduce the effect of potential confounders, the case and control groups were matched for their birth season and birthplace.

## Conclusions

The incidence rate of CH in East Azerbaijan province is 1 in 545 live births. History of CH in first degree relatives, parental consanguineous marriage, neonatal jaundice, and birth length are associated with an increased risk of CH. Moreover, in transient CH, in addition to the above risk factors, Betadine use by mothers and gestational age at birth also increased the disease risk.

## Data Availability

The datasets generated and/or analysed during the current study are not publicly available and the consent of the sponsoring organization is required. It is available from the corresponding author on reasonable request.

## Abbreviation

CH: Congenital hypothyroidism
